# Sleep duration affects the sequential change of body mass index and muscle strength: a contribution to dynapenic obesity

**DOI:** 10.1186/s12877-023-03857-7

**Published:** 2023-05-12

**Authors:** Zeyi Zhang, Jingjing Wang, Jingyi Wang, Bin Ma, Yuanmin Jia, Ou Chen

**Affiliations:** School of Nursing and Rehabilitation, Cheeloo College of Medicine, Shandong University, #44 West Wenhua Road, Jinan, 250012 China

**Keywords:** Sleep duration, Dynapenic obesity, Grip strength, Obesity, Mediation, Nonlinear

## Abstract

**Background:**

With aging, body mass index (BMI) increases and muscle strength declines, resulting in dynapenic obesity. It remains unknown whether and how sleep duration contributes to the sequence of BMI and muscle strength change in the progression of dynapenic obesity.

**Methods:**

Data were derived from the first two waves of China Health and Retirement Longitudinal Study. Sleep duration was self-reported. BMI was calculated and grip strength (GS) was measured to reflect muscle strength. The effect of baseline sleep duration on the sequential change of BMI and GS was assessed using two mediation models considering the nonlinear associations between them. The moderating effect of metabolic disorder was also tested.

**Results:**

Totally 4986 participants aged ≥ 50 years (50.8% females) with complete information on variables were included. Baseline BMI fully mediated the nonlinear association between sleep duration and follow-up GS change, but baseline GS did not mediate between sleep duration and follow-up BMI change for older men and women. Short sleep duration positively affected BMI-induced GS change (*β* = 0.038; 95%*CI*, 0.015–0.074), while this favorable effect became nonsignificant for moderate sleep duration (*β* = 0.008; 95% *CI*, -0.003–0.024) and turned negative with prolonged sleep duration (*β* =  − 0.022; 95%*CI*, − 0.051 to − 0.003). This nonlinear mediation effect was more pronounced in older women who are relatively metabolically healthy at baseline.

**Conclusion:**

For older adults in China, the influence of sleep duration on BMI-induced GS change but not the GS-induced BMI change suggested the contribution of sleep duration to the sequential course in the progression of dynapenic obesity. Sleep duration deviated either above or below normal range may confer adverse impact on GS through BMI. Strategies addressing sleep and obesity jointly to improve muscle function and delay the progression of dynapenic obesity are required.

**Supplementary Information:**

The online version contains supplementary material available at 10.1186/s12877-023-03857-7.

## Introduction

Musculoskeletal aging begins in midlife [1] and results in impaired neuromuscular strength in older adults [2]. The prevalence of obesity also increases with aging because of fat accumulation and muscle mass loss [3, 4]. Thus an emerging term indicating the co-occurrence of low muscle strength and obesity, namely “dynapenic obesity”, was raised [5]. Dynapenic obesity impacts nearly 10% of the old people [5]. Furthermore, the cumulative adverse effects of dynapenic obesity on disability and mortality are much more than those of dynapenia or obesity alone [6–8]. Therefore, factors influencing the progression of single component (i.e. obesity or dynapenia) to dynapenic obesity are highlighted.

Recent cross-sectional studies displayed an association of sleep duration with sarcopenia obesity [9, 10], which is correlated with dynapenic obesity. In fact, sleep duration is a shared influencing factor for obesity and low muscle strength [11–15]. There is also evidence showing that obesity-related variables (i.e. BMI) are predictors of deteriorated muscle strength [16–18]. Therefore, sleep duration may play a role in the progression of dynapenic obesity through affecting obesity-mediated muscle strength change. Conversely, muscle strength can also be a predictor of weight change [19, 20], which implies a potential effect of sleep duration on muscle strength-mediated obesity in the progression of dynapenic obesity. Accordingly, it is still unknown whether sleep duration affects fat accumulation before muscle function or vice-versa in the progression of dynapenic obesity. Moreover, existing studies showed nonlinear associations of sleep duration with muscle strength and BMI [15, 21, 22]. Addressing the effect of sleep duration on the sequential change of BMI and GS considering their nonlinear associations will be beneficial for better formulation of intervention strategy and implementation timing in preventing dynapenic obesity.

Furthermore, abnormal sleep duration and BMI are more prevalent in elders with metabolic disorders than those who are metabolically healthy [23, 24]. Metabolism-related factors such as insulin resistance are reckoned as the mechanisms of declined muscle function [25]. In this case, metabolic status may moderate the progression that sleep duration links to dynapenic obesity.

This study aims to investigate whether sleep duration affect the sequential change of BMI and muscle strength considering their nonlinear associations. Meanwhile, we estimate the moderating role of metabolic disorder in the above mechanism association. Findings will enhance the understanding of how sleep duration contributes to the progression of dynapenic obesity, which is pressing given the exacerbated obesity and muscle function problems during COVID-19 and their far-reaching impact [26, 27].

## Methods

### Study population

The China Health and Retirement Longitudinal Study (CHARLS) is a nationally representative survey sampled community-dwellers aged ≥ 45 years from 28 provinces in China, with a response rate of 80.5% [28]. The baseline data including demographics, health status and functioning, physical measures, and blood biochemical indicators were collected from 17,708 respondents in 2011. Participants have been followed biennially. The details about the design of CHARLS were published elsewhere [28].

**Fig. 1:**
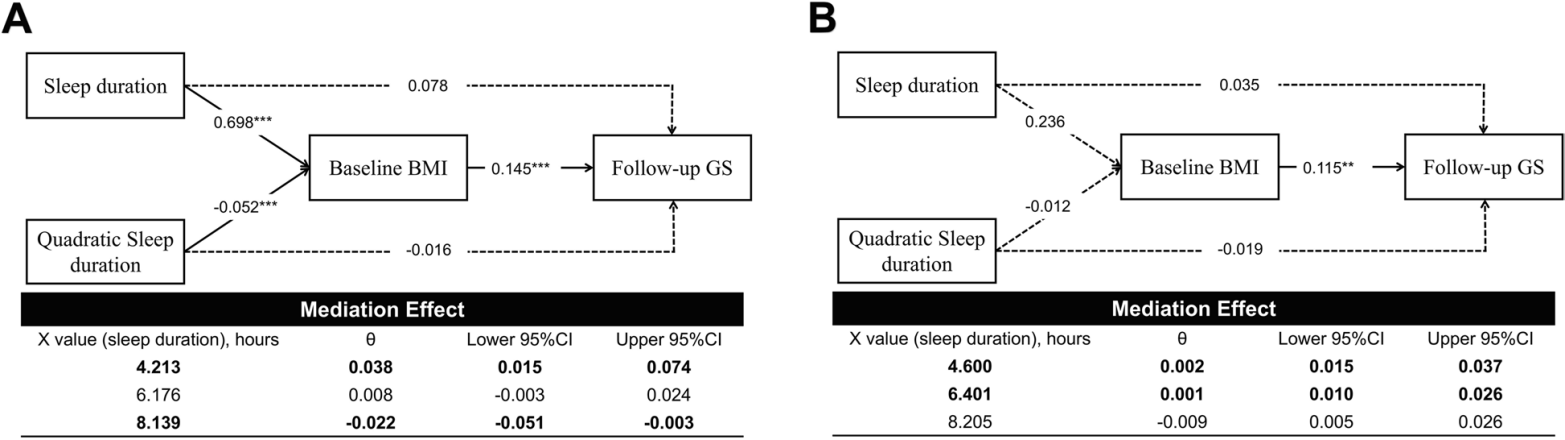
Nonlinear mediation models using baseline BMI as the mediator for men (**A**) and women (**B**) BMI, body mass index; GS, grip strength; CI, confidence interval; ^***^P < 0.001, ^**^P < 0.01. The solid line represents the statistically significant associations at *P* < 0.05 level, while the dashed line represents that the associations are not statistically significant at *P* < 0.05 level. The instantaneous indirect effect (θ) was estimated at M-1SD, M, and M + 1SD of sleep duration. If the CIs did not include 0, then the mediation effect of the mediation variable is significant. Models are adjusted for baseline GS, age, educational level, marital status, smoking status, drinking status, depression level, number of medical diagnosis, sleep quality, and use of hypnotics at baseline

Our study used longitudinal data from the first two waves of the CHARLS (i.e. wave 1, 2011 and wave 2, 2013). Participants were included in the current study if they: (1) aged ≥ 50 years at CHARLS baseline; (2) had complete baseline and follow-up data on sleep duration, BMI, and grip strength; (3) had not been diagnosed with cognition-related diseases (e.g. dementia and Alzheimer's disease) at baseline. We excluded participants who were missing on covariates (*n* = 2957). Ultimately, 4986 older adults were included (Fig. 1). Participants who are not included in this study were younger, had less smokers and married individuals, and had higher education levels, less comorbidities, lower depression level, longer sleep duration and higher baseline BMI (Table S1). Written informed consent was obtained from all participants. This research was approved by the Biomedical Ethics Review Committee of Peking University (approval number IRB00001052–11,015).

### Sleep duration

Nighttime sleep duration at baseline was obtained using the question “During the past month, how many hours of actual sleep did you get at night (average hours for one night)?” This sleep duration question is adapted from the Pittsburgh Sleep Quality Index, which has been validated in previous studies [29]. The continuous sleep duration was used for analyses.

### Muscle strength

Handgrip strength is a simple but well-established indicator of overall muscle strength. At both baseline and follow-up, grip strength was determined using a hand grip dynamometer (YuejianTM WL-1000 dynamometer) twice each hand. The maximum of the four measurements was used. Values of 0 or those above 100 kg were considered invalid.

### Obesity and metabolic indexes

Height was measured with a stadiometer (without shoes) to the nearest 0.1 cm; weight was measured with digital scales (minimal clothing, without shoes) to the nearest 0.1 kg. BMI, as an indicator of general obesity, was calculated using weight (kg) divided by squared height (m^2^). Waist circumference was measured horizontally around the subject at the level of navel while participants held their breath at the end of an exhalation.

At baseline, blood samples were taken after an overnight fast. Systolic blood pressure (SBP) and diastolic blood pressure (DBP) were measured using an automated electronic sphygmomanometer (OMRON™ HEM-7200) and calculated as the mean of three measurements at 45-s intervals. Metabolic disorders and their cut-off values were selected according to the criteria of metabolic syndrome [30]: SBP ≥ 140 mmHg and/or DBP ≥ 90 mmHg; triglyceride level ≥ 1.7 mmol/L; fasting blood high-density lipoprotein cholesterol level < 40 mg/dl for males or < 50 mg/dl for females; and fasting blood glucose ≥ 100 mg/dl. The number of metabolic disorders meeting the above criteria was calculated and used in the moderating analyses.

### Covariates

Covariates were chosen for inclusion based on their established relations with sleep and muscle strength. Variables included age, sex, educational level, marital status, smoking status, drinking status, depression level, number of self-reported chronic diseases, sleep quality and use of hypnotics. Details were demonstrated in the supplement.

### Statistical analysis

Sex differences have been observed in grip strength. Therefore, the current analyses were stratified by sex. Descriptive statistics (frequencies, percentages, means, and standard deviations (SD)) were presented to describe characteristics of the sample.

To examine the contribution of sleep duration to the progression of dynapenic obesity in older adults, we established two nonlinear mediation models. Specifically, given the nonlinear association of sleep duration with BMI and GS reported in the literature, we firstly verified the curve relationship by restricted cubic spline regression. Then, the nonlinear mediation models with sleep duration as the independent variable were constructed using MEDCURVE macro [31]. Such nonlinear mediation models are more suitable for this study, because the nonlinear relationships between variables suggest that the mediating effect may change with different values of independent variable, which will be obscured in traditional linear mediation analyses. In Model 1, BMI measured at baseline was the mediator and GS measured at follow-up was the dependent variable; while in the other model (Model 2), GS measured at baseline was the mediator and BMI measured at follow-up was the dependent variable. This allows us to test whether sleep duration affects the sequence of BMI and GS change. The mediation Model 1 adjusted for covariates and GS measured at baseline, so that the mechanism of sleep duration on BMI-induced GS changes was examined. Similarly, the mediation Model 2 adjusted for covariates and BMI measured at baseline, so that the mechanism of sleep duration on GS-induced BMI changes was examined. The mediating effect in these nonlinear associations was called the instantaneous indirect effect (θ). We estimated θ and the 95% confidence intervals (CIs) at short, moderate, and long sleep duration (i.e. mean sleep duration-1SD, mean, and mean sleep duration + 1SD, respectively), where θ changes with different levels of sleep duration. If the CIs did not include 0, then the mediation effect of the mediation variable is significant [31].

Furthermore, we assessed the moderated mediation effect using the PROCESS macro [32]. Based on the above mediation models, quadratic sleep duration was treated as the independent variable, and the number of metabolic disorders at baseline was treated as the moderator. We assessed the moderating effect at low, moderate, and high level of metabolic disorders, which is corresponding to the Mean-1SD, Mean, and Mean + 1SD of the number of metabolic disorders. Sleep duration and the interaction between sleep duration and number of metabolic disorder were additional control variables.

Several sensitivity analyses were performed. Firstly, there is evidence showing a nonlinear association between BMI and GS [19]. Therefore, we assessed the nonlinear mediation of sleep duration, BMI and GS. Besides, waist circumference or abdominal obesity was closely correlated to dynapenia [33, 34], of which the mechanism appears to be different from that of BMI. Hence, we conducted another two nonlinear mediation analyses (Model 3) using waist circumference instead of BMI to examine the contribution of sleep duration to the dynapenia abdominal obesity phenotype. Finally, we additionally adjusted for disability at baseline in all analyses. Data were analyzed using SPSS 25.0 and R 4.1.2. Two-tailed *P* value < 0.05 was considered significant.

## Results

### Characteristics of the participants

The characteristics of the 4986 participants (50.8% female) included in this study are shown in Table 1. The mean age was 62.4 ± 7.8 years for males and 61.5 ± 7.7 years for females. The average nighttime sleep duration was 6.4 ± 1.8 h and 6.2 ± 2.0 h, respectively. During the follow-up, the mean value of BMI increased from 22.8 to 23.0 kg/m^2^ for males and from 23.8 to 24.1 kg/m^2^ for females, while the mean value of GS decreased from 38.4 to 37.3 kg and from 26.3 to 25.4 kg, respectively. The number of metabolic disorders at baseline was higher in females (mean number 1.9) than in males (mean number 1.5).

**Table 1:** Characteristics of included participants by sex

Characteristics	Males (*n* = 2452)	Females (*n* = 2534)
Age (y), mean ± SD	62.4 ± 7.8	61.5 ± 7.7
Educational level, mean ± SD	3.7 ± 1.8	2.5 ± 1.7
Marital status (non-married) ^a^, n (%)	279 (11.4)	507 (20.0)
Smoking status (non-smokers), n (%)	594 (24.2)	2317 (91.4)
Drinking status (non-drinkers), n (%)	1369 (55.8)	2347 (92.6)
Medical diagnosis ^b^, mean ± SD	1.3 ± 1.4	1.5 ± 1.4
Depression level ^c^, mean ± SD	7.5 ± 5.6	9.6 ± 6.6
Sleep quality (poor), n (%)	399 (16.3)	576 (22.7)
Hypnotics (yes), n (%)	8 (0.3)	17 (0.7)
Baseline BMI (kg/m^2^), mean ± SD	22.8 ± 3.7	23.8 ± 4.0
Baseline GS (kg), mean ± SD	38.4 ± 8.9	26.3 ± 7.3
Follow-up BMI (kg/m^2^), mean ± SD	23.0 ± 3.5	24.1 ± 3.9
Follow-up GS (kg), mean ± SD	37.3 ± 9.0	25.4 ± 7.3
Baseline metabolic disorders, mean ± SD	1.5 ± 1.1	1.9 ± 1.2
Baseline sleep duration (hs), mean ± SD	6.4 ± 1.8	6.2 ± 2.0

*SD* standard deviation, *BMI* body mass index, *GS* grip strength

^a^Marital status was dichotomized into married and non-married (including married but not living with spouse, separated, divorced, widowed, and never married)

^b^Medical diagnosis was calculated as the number of having the following diseases: hypertension, dyslipidemia, diabetes, cancer, chronic lung diseases, liver diseases, heart diseases, stroke, kidney diseases, digestive disease, psychiatric problems, arthritis, and asthma

^c^Depression was measured with the ten-item Center for Epidemiologic Studies Depression Scale (CES-D10), excluding one item that assessed sleep quality

#### Nonlinear mediation models

The RCS regression demonstrated inversed U-shaped associations of baseline sleep duration with BMI and GS at baseline and follow-up for both males and females (all *P*_non-linear_ < 0.001, Figure S2-S3). Therefore, nonlinear mediation models are required to investigate the mechanisms of sleep duration on dynapenic obesity.

In the mediation Model 1 (Fig. 1A), for females, quadratic sleep duration affected baseline BMI (*β* = -0.052, *P* < 0.001), and BMI affected GS change at follow-up (*β* = 0.145, *P* < 0.001). There was no significant direct association between sleep duration and GS change (*β* = 0.078, *P* = 0.807). Therefore, BMI fully mediated the nonlinear relationship between sleep duration and GS change. The instantaneous indirect effect (θ) indicated that sleep duration positively affected GS change through BMI among those who reported short sleep duration (*β* = 0.038; 95% *CI*, 0.015–0.074). The mediating effect of BMI decreased with sleep duration from short to moderate (*β* = 0.008; 95% *CI*, -0.003–0.024). Conversely, longer sleep duration negatively affected GS change through BMI (*β* =  − 0.022; 95% *CI*, − 0.051 to − 0.003). For older males (Fig. 1B), similar decreasing tendency with more positive values of mediating effect was observed with longer sleep duration (Model 1).

**Fig. 2:**
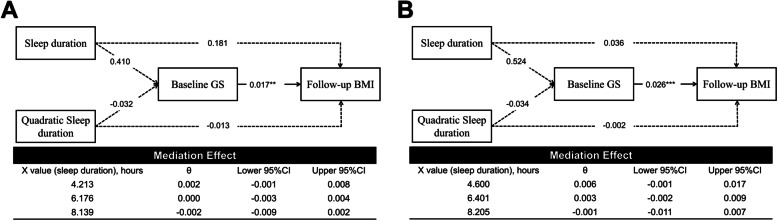
Nonlinear mediation models using baseline GS as the mediator for men (**A**) and women (**B**). BMI, body mass index; GS, grip strength; CI, confidence interval; ^***^P < 0.001. The solid line represents the statistically significant associations at *P* < 0.05 level, while the dashed line represents that the associations are not statistically significant at *P* < 0.05 level. The instantaneous indirect effect (θ) was estimated at M-1SD, M, and M + 1SD of sleep duration. If the CIs did not include 0, then the mediation effect of the mediation variable is significant. Models are adjusted for baseline BMI, age, educational level, marital status, smoking status, drinking status, depression level, number of medical diagnosis, sleep quality, and use of hypnotics at baseline

The mediation model 2 (Fig. 2) showed that sleep duration had no effect on either baseline GS or BMI changes for males and females. There was an association between baseline GS and BMI change at follow-up (*β* = 0.017, *P* = 0.006 and *β* = 0.026, *P* < 0.001 for males and females, respectively).

#### Moderated mediation effect

**Fig. 3:**
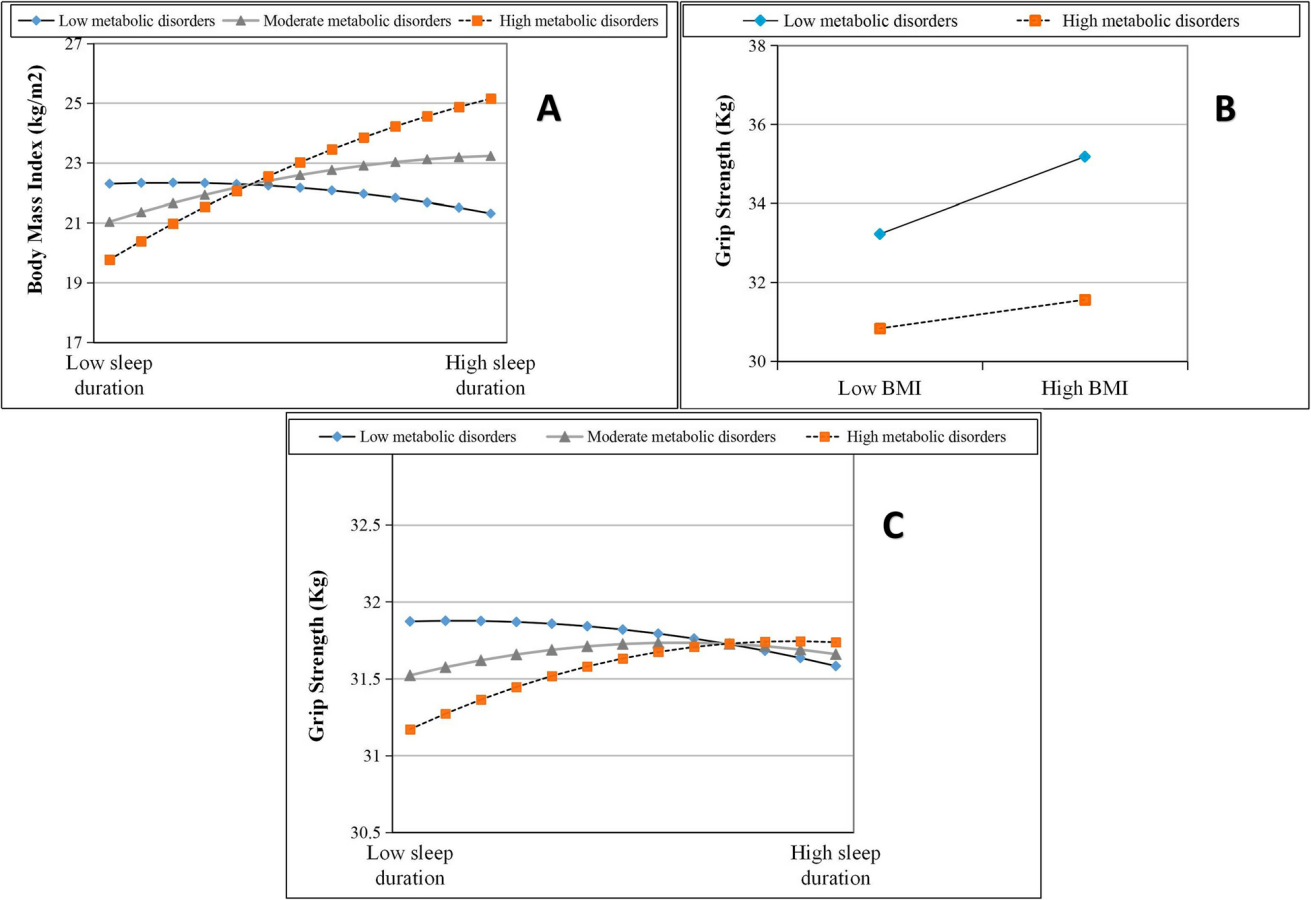
Moderating effect of baseline metabolic disorder on the nonlinear mediation (**A**, **B**) and direct effect (**C**). BMI, body mass index; GS, grip strength; models are adjusted for baseline BMI, age, educational level, marital status, smoking status, drinking status, depression level, number of medical diagnosis, sleep quality, and use of hypnotics at baseline

The moderating effect of metabolic disorder was examined only for the significant mediation models (Table 2). Results revealed that metabolic disorder regulated the nonlinear association between sleep duration and GS change (*β*_quadratic sleep duration*metabolic disorder_ = -0.009, *P* < 0.001) in elderly females. Figure 3A showed that when older women had more metabolic disorders, BMI increased more with longer sleep duration. Nevertheless, marginal significant interactions of BMI and metabolic disorder on GS change (β _baseline BMI*metabolic disorder_ = 0.051, *P* = 0.095) showed that increase in GS induced by higher BMI was not significant in women with more metabolic disorders (Fig. 3B). Therefore overall, older women with more metabolic disorders had lower GS levels (Fig. 3C). No significant moderating effect was found in males.

**Table 2:** The moderating effect of metabolic disorders in the nonlinear mediation models

Variables	Baseline body mass index*				Follow-up grip strength*			
	*β*	SE	*P*	95%*CI*	*β*	SE	*P*	95%*CI*
**Female**								
Linear sleep duration	0.366	0.175	0.036	0.023, 0.709	0.023	0.323	0.944	-0.610, 0.655
Quadratic sleep duration	-0.051	0.014	< 0.001	-0.078, -0.023	-0.015	0.026	0.553	-0.065, 0.035
Metabolic disorders	0.353	0.082	< 0.001	0.192, 0.515	-0.079	0.158	0.616	-0.388, 0.230
Linear sleep duration× Metabolic disorders	0.442	0.034	< 0.001	0.376, 0.509	0.060	0.069	0.384	-0.076, 0.196
Quadratic sleep duration× Metabolic disorders	-0.009	0.003	< 0.001	-0.014, -0.004	-0.004	0.005	0.453	-0.013, 0.006
Baseline body mass index					0.134	0.037	< 0.001	0.061, 0.206
Baseline body mass index× Metabolic disorders					-0.051	0.031	0.095	-0.111, 0.009
**Male**								
Linear sleep duration	0.040	0.180	0.823	-0.313, 0.393	0.019	0.408	0.964	-0.782, 0.819
Quadratic sleep duration	-0.012	0.014	0.386	-0.039, 0.015	-0.018	0.032	0.570	-0.080, 0.044
Metabolic disorders	0.360	0.090	< 0.001	0.183, 0.538	-0.007	0.217	0.973	-0.433, 0.418
Linear sleep duration× Metabolic disorders	0.316	0.031	< 0.001	0.256, 0.376	0.007	0.074	0.927	-0.139, 0.153
Quadratic sleep duration× Metabolic disorders	-0.004	0.003	0.212	-0.009, 0.002	-0.006	0.006	0.392	-0.018, 0.007
Baseline body mass index					0.123	0.047	0.009	0.031, 0.215
Baseline body mass index× Metabolic disorders					-0.036	0.039	0.359	-0.112, 0.041

*SE* standard error, *CI* confidence interval

*The moderated mediation model adjusted for baseline grip strength, age, educational level, marital status, smoking status, drinking status, depression level, number of medical diagnosis, sleep quality, and use of hypnotics at baseline

#### Sensitivity analyses

Considering the nonlinear association of BMI with GS (Figure S4), for both males and females, baseline BMI displayed a full nonlinear mediating effect between sleep duration and GS change at follow-up. Similar to the main analyses, the positive mediating effect of BMI decreased and turned negative with longer sleep duration (Table S2). When waist circumference was adopted to examine the mechanism of sleep duration on dynapenia abdominal obesity, certain pathways lost statistical significance, demonstrating some differences from the main analysis of BMI as the mediator (Table S3). Finally, additional adjustment of the baseline disability in the analyses had little effect on the results (Table S4-S5).

## Discussion

This study showed that BMI fully mediated the nonlinear association between sleep duration and subsequent GS change in older men and women. The mediating effect was positive for elders with relatively shorter sleep duration and became negative for those with prolonged sleep duration. There was no GS-mediated effect between sleep duration and BMI change. Findings suggested that sleep duration has an effect on the sequential change of BMI and GS, and thus contributes to the progression of single component to dynapenic obesity in older adults. Additionally, the adverse effects of longer sleep on BMI and GS change were more pronounced in older women who were metabolically healthy at baseline.

The key finding of this study is that sleep duration contributes to the sequential change of BMI and GS, which adds to the sparse literature regarding the effect of sleep duration on obesity, muscle strength and dynapenic obesity. A recent study suggested that the effect of objectively measured nighttime sleep duration on GS was independent of BMI [35]. However, in a sample with characteristics similar to our study population, sleep duration was linked to sarcopenia obesity [9]. Nevertheless, the above studies adopted a cross-sectional study design, which may preclude further exploration of how sleep duration affect the time course of dynapenic obesity. Our study applied longitudinal data and formal mediation analysis, which is unique from prior studies. Results showed that sleep duration affect BMI-induced GS change but not the vice-versa. That means sleep duration influence obesity-related change before muscle strength change in the progression of dynapenic obesity. This finding is rationale given the reported association of sleep duration with insulin resistance and systematic inflammation [36, 37], which are involved in obesity and sarcopenia. Sleep duration is also associated with metabolism (e.g. leptin level) and energy intake, which will lead to fat accumulation and obesity [38]. Moreover, intramuscular fat can induce loss of muscle mass and function through oxidation stress [39, 40]. Although we also found that GS at baseline was related to BMI change at follow-up, which is similar to the results in previous studies [19, 20], this association should be further verified given the few plausible mechanisms by which GS could affect BMI change and the potential reverse causation.

Nonlinear relationships of sleep duration with BMI and GS have been demonstrated previously [22, 41], and were also supported by our study. Therefore, the varied mediating effect according to sleep duration should be taken into account. Indeed, we observed that different sleep duration has different influence on the progression of dynapenic obesity. For those with short or moderate nighttime sleep, sleep duration positively affected subsequent GS change through BMI. This can be interpreted as the effect of sleep duration on the “healthy obese” phenotype, which is proposed as the co-occurrence of higher GS and obesity [42] and may be the early stage of dynapenic obesity. By contrast, prolonged sleep negatively affected BMI-induced subsequent GS change, leading to reduced BMI and GS. This can be deemed as manifestations of frailty (i.e. weight loss and low handgrip strength) [43], indicating the influence of sleep duration on the later outcome of dynapenic obesity. In the present study, the nonlinear association between BMI and GS change was explored as well. Results further our understanding that longer sleep duration may lead to excessive BMI and decreased GS, contributing to the occurrence of dynapenic obesity. Therefore, the influence of sleep duration seems to run through the whole process of developing dynapenic obesity. These findings provide supporting evidence on prudently pursuing long sleep for sleeping interventions among older adults.

We also tested waist circumference as the mediator. Different from BMI, which integrates lean body mass and fat mass, waist circumference is more presentative for fat mass in the abdomen. Results demonstrated a similar decreasing mediating effect except for prolonged sleep duration. This finding implied that muscle mass and fat mass may be independent pathways through which prolonged sleep duration affects GS change. This can also be cited to explain the sex difference in the present study that some associations are not significant in men. As evidence shows, men and women have a larger proportion of muscle mass and fat mass, respectively [44, 45].

Furthermore, we observed metabolic health modify the mediation in elder women. Those had fewer metabolic disorders are in favor of relatively short sleep duration, which showed increased BMI and GS. Such “metabolically healthy obesity” phenotype has been reported to have better cardiovascular outcomes than “metabolically unhealthy obesity” [46]. Our study extends the benefit of “metabolically healthy obesity” phenotype to the domain of muscle function. However, such favorable effect diminished with longer sleep duration. As for those with more metabolic disorders, BMI rose steeply with longer sleep duration and exceeded normal thresholds. Nevertheless, GS did not improve significantly with increased BMI, giving rise to the overall lower GS levels and the development of dynapenic obesity. Therefore, interventions (e.g. behavioral interventions on sleep or weight) are required to prevent or intervene with metabolic disorders to delay muscle strength decline and the progression of dynapenic obesity [47, 48].

Our findings suggested that sleep duration interventions may delay the progression of dynapenic obesity by interfering with the cyclic process of BMI-induced muscle strength change. Additional benefits will be gained especially when combining with weight control. This will provide support for combined lifestyle intervention [49, 50], which is increasingly appreciated. Besides, adverse effect of sleep duration and BMI deviated either above or below the normal range is concerning. Especially in the context of COVID-19 pandemic, older people are isolated at home and may be prone to altering sleep pattern and accumulating fat mass [26, 27]. Thus a combined intervention that simultaneously observes and controls sleep duration and BMI within the normal range will have profound effect not only on cardiovascular outcomes [51] but also on delaying the progression of dynapenic obesity in general older adults.

### Strengths and limitations

This study demonstrated the effect of sleep duration on the sequential change of BMI and GS in the older population for the first time. The novel nonlinear mediation analyses showed different mechanism effects of BMI under different sleep duration in the progression of dynapenic obesity. However, some limitations should be acknowledged. Firstly, sleep duration was assessed by self-reported methods, and recall bias is a concern. However, a good correlation between subjective sleep duration and objective measures was reported [52]. In addition, in such a large sample of “healthy” community residents, it is more realistic to obtain self-reported sleep duration. Similarly, objective muscle and fat indicators are not accessible in this large sample. Our study was also limited by the focus on sleep duration only. Other sleep parameters (e.g. quality and timing) have been linked to multiple outcomes and remain understudied for their association with GS [14]. However, this study took interest in sleep duration because it is readily available and is easy to intervene with. Thirdly, although confounders have been controlled, some factors were not fully considered (e.g. diet). We attempted to adjust for the use of sleeping pills by single simple question due to the lack of medication records. Another limitation is the short follow-up of years. Finally, our sample was characterized by community-dwelling older adults in China and was relatively unhealthy compared with those excluded. Whether these findings are applicable to other populations remains to be examined in large prospective studies with longer follow-up and more comprehensive measures.

## Conclusion

Sleep duration affected the BMI-induced GS change but not the GS-induced BMI change in older adults, thus contributing to the progression of dynapenic obesity. This BMI-mediated effect is nonlinear, which decreased and turned negative with prolonged sleep duration, especially for older women with less metabolic disorders. These results disclosed that sleep duration deviated either above or below normal range may translate to adverse influence on GS through BMI. Moreover, strategies aiming at addressing sleep and obesity jointly (e.g. combined behavioral intervention) to delay the development of dynapenic obesity are called for.

## Supplementary Information

**Additional file 1.**

## Data Availability

The data used and analyzed in the current study are available from the website http://charls.pku.edu.cn/

## Abbreviations

BMI: Body mass index
GS: Grip strength
CHARLS: China Health and Retirement Longitudinal Study
CI: Confidence interval
SD: Standard deviation
SBP: Systolic blood pressure
DBP: Diastolic blood pressure
