# Supplementary material for: Sleep duration affects the sequential change of body mass index and muscle strength: a contribution to dynapenic obesity
Source: BMC Geriatr. 2023 May 12;23:288. doi: 10.1186/s12877-023-03857-7 (PMC10177716; doi:10.1186/s12877-023-03857-7)
Supplement: Supplementary file 1 — Additional file 1. [file 12877_2023_3857_MOESM1_ESM.docx]

**Supplement**

**Sleep Duration Contributes to Dynapenia Obesity: the Sequence of Body Mass Index and Muscle Strength Change**

Zeyi Zhang^a^ (M.D. Zhzeyi@outlook.com), Jingjing Wang^a^ (M.D. 304254357@qq.com), Jingyi Wang^a^ (M.D. 449031427@qq.com), Bin Ma^a^ (M.D. 1543001572@qq.com), Yuanmin Jia^a^ (M.D. 3301099805@qq.com), Ou Chen^a^*(PhD. [chenou@sdu.edu.cn](mailto:chenou@sdu.edu.cn))

^a^ School of Nursing and Rehabilitation, Cheeloo College of Medicine, Shandong University, #44 West Wenhua Road, Jinan 250012, China.

*Correspondence: Ou Chen

School of Nursing and Rehabilitation, Cheeloo College of Medicine, Shandong University, #44 West Wenhua Road, Jinan 250012, China

Tel: 86+15990991181; Fax: 0531-88382201

Email: [chenou@sdu.edu.cn](mailto:chenou@sdu.edu.cn)

**Contents:**

eMethod 1 Covariates

eMethod 2 Sensitivity analyses

Table S1 Characteristics of participants included and excluded

Table S2 Sensitivity analyses considering the nonlinear association between BMI and GS

Table S3 Sensitivity analyses regarding the contribution of sleep duration to the dynapenia abdominal obesity phenotype in Model 3

Table S4 Sensitivity analyses additionally adjusted for disability at baseline

Table S5 The moderating effect of metabolic disorders in the nonlinear mediation models additionally adjusted for disability at baseline

Figure S1 Flow chart of including participants

Figure S2 Nonlinear associations between baseline sleep duration and baseline BMI (A), baseline sleep duration and follow-up BMI (B), baseline sleep duration and baseline GS (C), and baseline sleep duration and follow-up GS (D) for females.

Figure S3 Nonlinear associations between baseline sleep duration and baseline BMI (A), baseline sleep duration and follow-up BMI (B), baseline sleep duration and baseline GS (C), and baseline sleep duration and follow-up GS (D) for males.

Figure S4 Nonlinear associations between baseline BMI and follow-up GS for females (A) and males (B).

**eMethod 1 Covariates**

Data on sociodemographic variables included age, sex, educational attainment (assessed as a continuous variable using 11 levels), and marital status (dichotomized as married/not). Smoking status and drinking status at baseline interview were grouped into current/former or never. Sleep quality was assessed with the question: “How often do you feel the quality of your sleep is bad during sleep last week?” Response was categorized into “good/fair” (<1 day, 1-2 or 3-4 days) and “poor” (5-7 days). Information on [hypnotics](https://www.youdao.com/w/hypnotics/#keyfrom=E2Ctranslation) (yes/no) was assessed with the question: “Do you treat emotional or psychiatric problems by taking sleeping pills?” Participants were asked about medical diagnoses (hypertension, dyslipidemia, diabetes, cancer, chronic lung diseases, liver diseases, heart diseases, stroke, kidney diseases, digestive disease, psychiatric problems, arthritis, and asthma) at baseline, and the number of comorbidities was calculated. Depressive symptoms at baseline were measured with the Chinese version of the ten-item Center for Epidemiologic Studies Depression Scale (CES-D10), with higher scores indicating more depressive symptoms. Because an item in this scale overlapped with the sleep quality assessment question, it was excluded from the depression score calculation.

**eMethod 2 Sensitivity analyses**

Several previous studies showed that there is nonlinear association between BMI and GS. In our study, we did observe a significant nonlinear association between BMI and GS. However, the inflection point of the curve (around BMI of 27.8 kg/m2) is greater than the 85% threshold of population distribution (BMI of 26.3 and 27.7 kg/m2 for males and females). Besides, BMI and GS showed a linear association before the inflection point, while this trend tended to be gentle after the reflection point. Therefore, we assumed a linear relationship between BMI and GS change in the main analyses, while we tested a nonlinear relationship in the sensitivity analysis by specifying quadratic relationships among the three variables of sleep duration, BMI and GS in the nonlinear mediation models. That is to say, in the MEDCURVE macro, we set a quadratic association between BMI and GS in this sensitivity analyses instead of a linear association (as in the main analyses) in addition to the setting of quadratic associations between sleep duration and BMI, and sleep duration an GS.

Disability information was incomplete for some individuals. Therefore, to retain statistical efficiency, we additionally adjusted for baseline disability status for sensitivity analyses. The ADL (6 items) and IADL (5 items) scale were used to estimate each respondent’s ability during daily life. Disability (yes/no) was identified if the respondent answered “have difficulty to do” or “cannot do at all/unable to do” to one of the following 11 activities: eating, dressing, bathing, transferring in and out of bed, using the toilet, controlling urination and defecation, doing household chores, preparing meals, shopping, managing money, and taking medicine.

Table S1 Characteristics of participants included and excluded

| **Characteristics** | **Included (n=4986)** | **Excluded (n=12722)** | ***P* value** |
| --- | --- | --- | --- |
| Age (y), mean±SD | 61.9±7.7 | 57.9±10.8 | **<0.001** |
| Sex (male), n (%) | 2452 (49.2) | 6019 (47.4) | 0.079 |
| Educational level, mean±SD | 3.1±1.9 | 3.5±2.0 | **<0.001** |
| Marital status (non-married) ^a^, n (%) | 786 (15.8) | 2719 (21.4) | **<0.001** |
| Smoking status (non-smokers), n (%) | 2911 (58.4) | 7714 (61.4) | **<0.001** |
| Drinking status (non-drinkers), n (%) | 3716 (74.5) | 9453 (75.2) | 0.335 |
| Medical diagnosis ^b^, mean±SD | 1.4±1.4 | 1.3±1.4 | **<0.001** |
| Depression level ^c^, mean±SD | 8.6±6.3 | 8.3±6.4 | **0.024** |
| Sleep quality (poor), n (%) | 975 (19.6) | 2164 (19.7) | 0.934 |
| Hypnotics (yes), n (%) | 25 (0.5) | 62 (0.5) | 0.872 |
| Baseline BMI (kg/m^2^), mean±SD | 23.3±3.9 | 23.5±3.9 | **0.002** |
| Baseline GS (kg), mean±SD | 32.2±10.1 | 32.5±10.7 | 0.102 |
| Follow-up BMI (kg/m^2^), mean±SD | 23.6±3.8 | 24.0±3.8 | **<0.001** |
| Follow-up GS (kg), mean±SD | 31.3±10.1 | 33.1±10.7 | **<0.001** |
| Baseline metabolic disorders, mean±SD | 1.7±1.2 | 1.7±1.2 | 0.201 |
| Baseline sleep duration (hs), | 6.3±1.9 | 6.4±1.9 | **<0.001** |

SD, standard deviation; BMI, boy mass index; GS, grip strength

^a^ Marital status was dichotomized into married and non-married (including married but not living with spouse, separated, divorced, widowed, and never married).

^b^ Medical diagnosis was calculated as the number of having the following diseases: hypertension, dyslipidemia, diabetes, cancer, chronic lung diseases, liver diseases, heart diseases, stroke, kidney diseases, digestive disease, psychiatric problems, arthritis, and asthma.

^c^ Depression was measured with the ten-item Center for Epidemiologic Studies Depression Scale (CES-D10), excluding one item that assessed sleep quality.

Table S2 Sensitivity analyses considering the nonlinear association between BMI and GS ^a^

| **Variables** | **Baseline BMI** | |  | **Follow-up GS** | |  | **Instantaneous indirect effect (θ)^a^** |
| --- | --- | --- | --- | --- | --- | --- | --- |
|  | *β* | *P* |  | *β* | *P* |  |  |
| **Female** |  |  |  |  |  |  | X LowerCI θ UpperCI  **4.213 0.018 0.049 0.088**  6.176 -0.005 0.010 0.026  **8.139 -0.062 -0.027 -0.004** |
| Linear sleep duration | 0.699 | <0.001 |  | 0.070 | 0.826 |  |  |
| Quadratic sleep duration | -0.052 | <0.001 |  | -0.016 | 0.531 |  |  |
| Linear body mass index |  |  |  | 0.689 | <0.001 |  |  |
| Quadratic body mass index |  |  |  | -0.011 | 0.004 |  |  |
| **Male** |  |  |  |  |  |  | X LowerCI θ UpperCI  **4.598 0.005 0.021 0.053**  **6.401 0.003 0.014 0.033**  8.204 -0.014 0.007 0.032 |
| Linear sleep duration | 0.236 | 0.224 |  | 0.033 | 0.936 |  |  |
| Quadratic sleep duration | -0.012 | 0.432 |  | -0.019 | 0.548 |  |  |
| Linear body mass index |  |  |  | 0.475 | 0.013 |  |  |
| Quadratic body mass index |  |  |  | -0.007 | 0.054 |  |  |

BMI, body mass index; GS, grip strength; CI, confidence interval

^a^: The instantaneous indirect effect (θ) was calculated from the nonlinear mediation models, considering the nonlinear associations between sleep duration and BMI, sleep duration and GS, and BMI and GS. Models adjusted for baseline grip strength, age, educational level, marital status, smoking status, drinking status, depression level, number of medical diagnosis, sleep quality, and use of hypnotics.

Table S3 Sensitivity analyses regarding the contribution of sleep duration to the dynapenia abdominal obesity phenotype in Model 3 ^a^

| **Variables** | **Baseline WC** | |  | **Follow-up GS** | |  | **Instantaneous indirect effect (θ)^a^** |
| --- | --- | --- | --- | --- | --- | --- | --- |
|  | *β* | *P* |  | *β* | *P* |  |  |
| **Female** |  |  |  |  |  |  | X LowerCI θ UpperCI  **4.213 0.001 0.017 0.041**  6.176 -0.006 0.004 0.014  8.139 -0.031 -0.010 0.005 |
| Linear sleep duration | 1.172 | 0.052 |  | 0.112 | 0.724 |  |  |
| Quadratic sleep duration | -0.088 | 0.069 |  | -0.019 | 0.462 |  |  |
| Linear WC |  |  |  | 0.038 | <0.001 |  |  |
| **Male** |  |  |  |  |  |  | X LowerCI θ UpperCI  **4.598 0.006 0.024 0.050**  **6.401 0.008 0.020 0.039**  8.204 -0.004 0.016 0.040 |
| Linear sleep duration | 0.727 | 0.254 |  | 0.024 | 0.954 |  |  |
| Quadratic sleep duration | -0.023 | 0.648 |  | -0.019 | 0.551 |  |  |
| Linear WC |  |  |  | 0.046 | <0.001 |  |  |

WC, waist circumference; GS, grip strength; CI, confidence interval;

^a^: The mediation model adjusted for baseline grip strength, age, educational level, marital status, smoking status, drinking status, depression level, number of medical diagnosis, sleep quality, and use of hypnotics.

Table S4 Sensitivity analyses additionally adjusted for disability at baseline ^a^

| **Variables** | **Baseline BMI** | |  | **Follow-up GS** | |  | **Instantaneous indirect effect (θ)^a^** |
| --- | --- | --- | --- | --- | --- | --- | --- |
|  | *β* | *P* |  | *β* | *P* |  |  |
| **Female** |  |  |  |  |  |  | X LowerCI θ UpperCI  **4.022 0.018 0.040 0.082**  6.020 -0.001 0.010 0.027  **8.018 -0.051 -0.021 -0.002** |
| Linear sleep duration | 0.759 | <0.001 |  | 0.188 | 0.598 |  |  |
| Quadratic sleep duration | -0.057 | 0.001 |  | -0.024 | 0.421 |  |  |
| Linear BMI |  |  |  | 0.135 | <0.001 |  |  |
| **Male** |  |  |  |  |  |  | X LowerCI θ UpperCI  **4.361 0.004 0.025 0.061**  **6.251 0.001 0.014 0.036**  8.204 -0.020 0.003 0.030 |
| Linear sleep duration | 0.381 | 0.116 |  | -0.220 | 0.655 |  |  |
| Quadratic sleep duration | -0.022 | 0.241 |  | -0.002 | 0.958 |  |  |
| Linear BMI |  |  |  | 0.135 | 0.011 |  |  |

BMI, body mass index; GS, grip strength; CI, confidence interval

^a^: The mediation model adjusted for baseline grip strength, age, educational level, marital status, smoking status, drinking status, depression level, number of medical diagnosis, sleep quality, use of hypnotics, and disability status at baseline.

Table S5 The moderating effect of metabolic disorders in the nonlinear mediation models additionally adjusted for disability at baseline ^a^

| **Variables** | **Baseline body mass index** | | | |  | **Follow-up grip strength** | | | |
| --- | --- | --- | --- | --- | --- | --- | --- | --- | --- |
|  | *β* | SE | *P* | 95%*CI* |  | *β* | SE | *P* | 95%*CI* |
| **Female** |  |  |  |  |  |  |  |  |  |
| Linear sleep duration | 0.405 | 0.199 | 0.041 | 0.016, 0.794 |  | 0.078 | 0.362 | 0.829 | -0.631, 0.787 |
| Quadratic sleep duration | -0.055 | 0.016 | <0.001 | -0.087, -0.024 |  | -0.018 | 0.029 | 0.541 | -0.075, 0.039 |
| Metabolic disorders | 0.317 | 0.094 | <0.001 | 0.133, 0.501 |  | -0.109 | 0.177 | 0.537 | -0.456, 0.238 |
| Linear sleep duration*  Metabolic disorders | 0.480 | 0.040 | <0.001 | 0.401, 0.559 |  | 0.069 | 0.081 | 0.394 | -0.090, 0.229 |
| Quadratic sleep duration*  Metabolic disorders | -0.010 | 0.003 | <0.001 | -0.016, -0.004 |  | -0.036 | 0.034 | 0.289 | -0.103, 0.031 |
| Baseline body mass index |  |  |  |  |  | 0.123 | 0.041 | 0.003 | 0.043, 0.204 |
| Baseline body mass index*  Metabolic disorders |  |  |  |  |  | -0.036 | 0.034 | 0.289 | -0.103, 0.031 |
| **Male** |  |  |  |  |  |  |  |  |  |
| Linear sleep duration | 0.149 | 0.224 | 0.506 | -0.290, 0.588 |  | -0.234 | 0.495 | 0.637 | -1.205, 0.737 |
| Quadratic sleep duration | -0.022 | 0.018 | 0.201 | -0.057, 0.012 |  | -0.001 | 0.039 | 0.986 | -0.077, 0.075 |
| Metabolic disorders | 0.377 | 0.118 | 0.001 | 0.146, 0.608 |  | -0.054 | 0.276 | 0.846 | -0.595, 0.487 |
| Linear sleep duration*  Metabolic disorders | 0.353 | 0.041 | <0.001 | 0.273, 0.432 |  | -0.018 | 0.097 | 0.851 | -0.209, 0.172 |
| Quadratic sleep duration*  Metabolic disorders | -0.003 | 0.004 | 0.383 | -0.011, 0.004 |  | -0.013 | 0.008 | 0.128 | -0.029, 0.004 |
| Baseline body mass index |  |  |  |  |  | 0.157 | 0.058 | 0.007 | 0.043, 0.272 |
| Baseline body mass index*  Metabolic disorders |  |  |  |  |  | -0.041 | 0.052 | 0.435 | -0.143, 0.061 |

SE, standard error; CI, confidence interval

^a^: The moderated mediation model adjusted for baseline grip strength, age, educational level, marital status, smoking status, drinking status, depression level, number of medical diagnosis, sleep quality, use of hypnotics, and disability status at baseline.

17708 individuals were interviewed in the first wave of CHARLS in 2011

15770 individuals were successfully re-interviewed in the second wave in 2013

7827 individuals were excluded because:

1. Aged <50 years at CHARLS baseline (n=3971);
2. had incomplete baseline and follow-up data on sleep duration, BMI, and grip strength (n=4640);
3. had been diagnosed with cognition-related diseases (n=116)

7943 individuals met the inclusion criteria

2957 individuals were excluded because missing on covariates

4986 individuals were included in this study

Figure S1 Flow chart of including participants


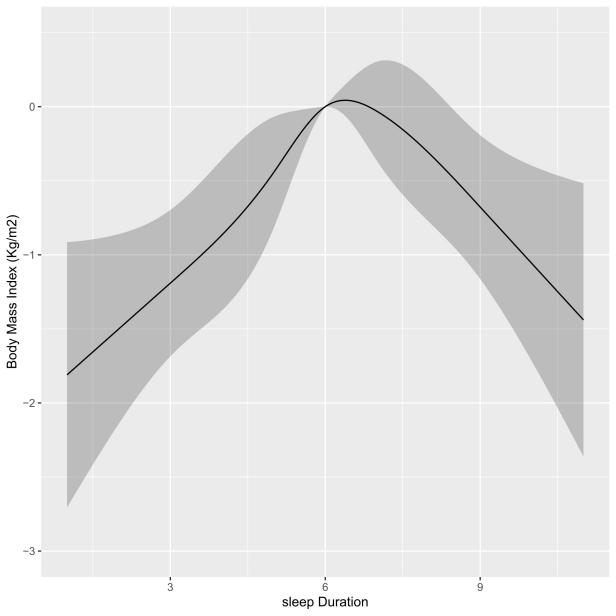

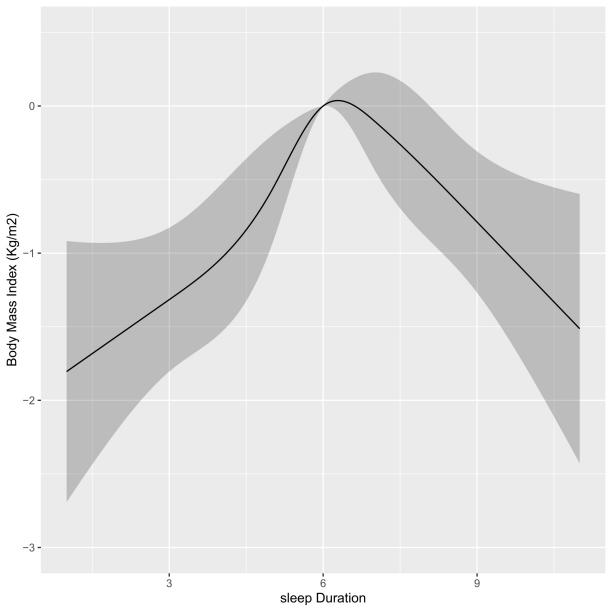


**D**

**C**

**B**

**A**


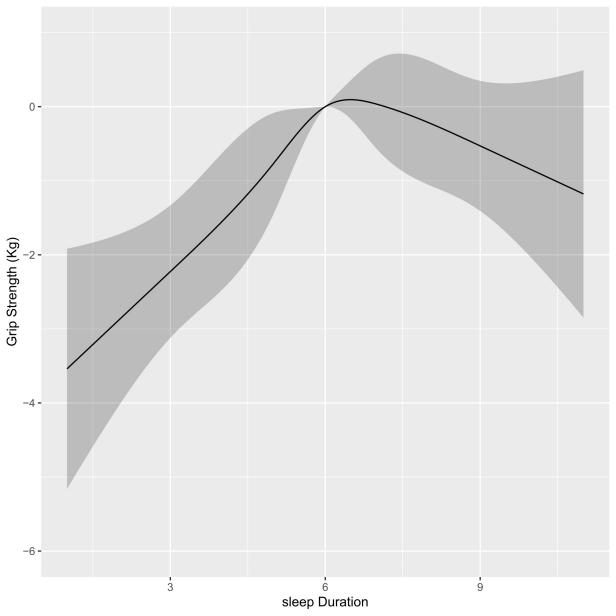

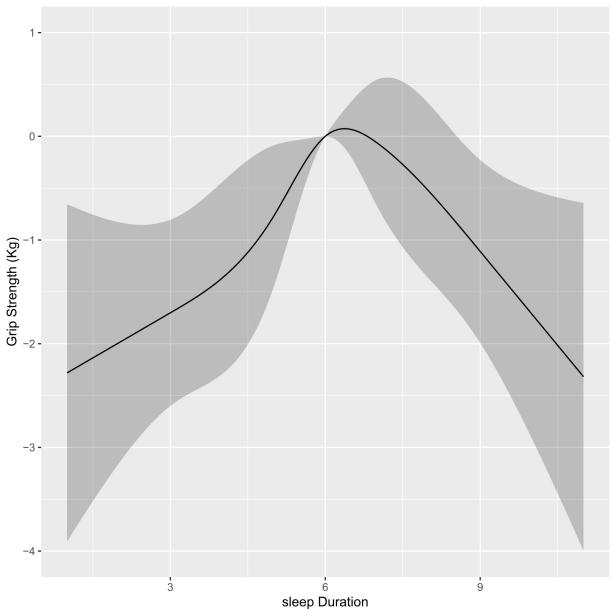


Figure S2 Nonlinear associations between baseline sleep duration and baseline BMI (A), baseline sleep duration and follow-up BMI (B), baseline sleep duration and baseline GS (C), and baseline sleep duration and follow-up GS (D) for females.


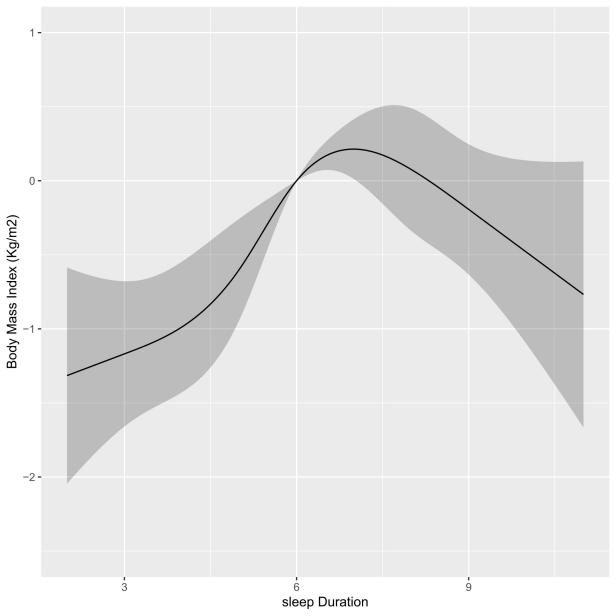

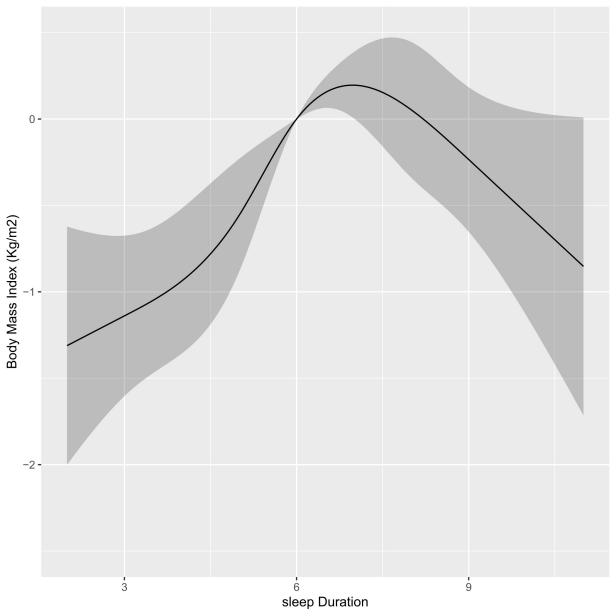


**D**

**B**

**A**

**C**


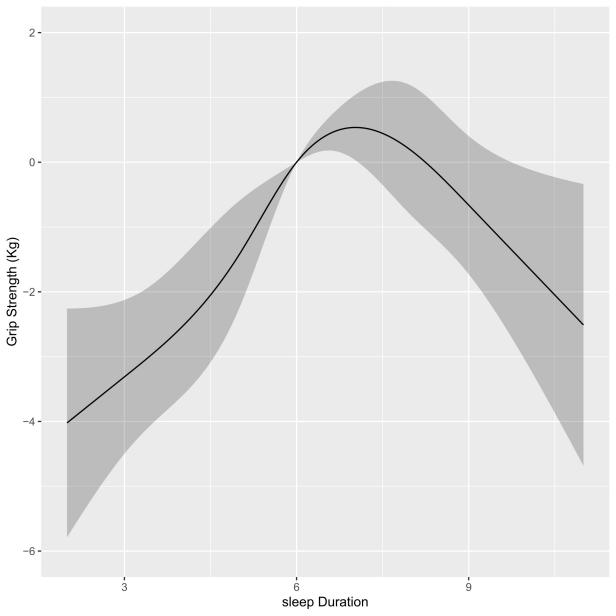

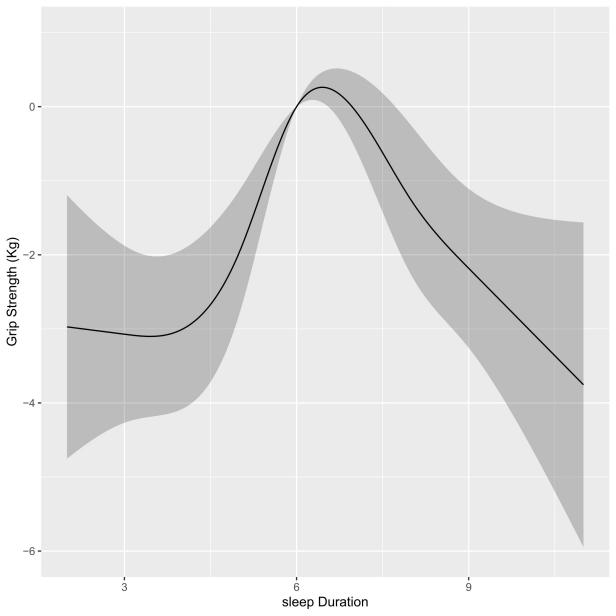


Figure S3 Nonlinear associations between baseline sleep duration and baseline BMI (A), baseline sleep duration and follow-up BMI (B), baseline sleep duration and baseline GS (C), and baseline sleep duration and follow-up GS (D) for males.


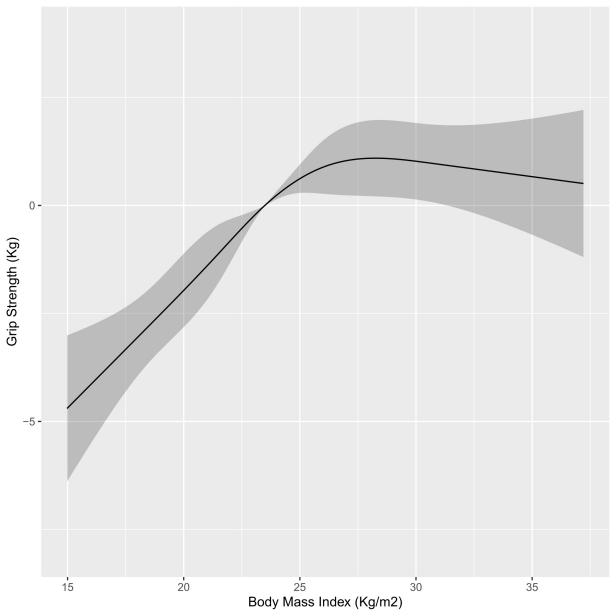

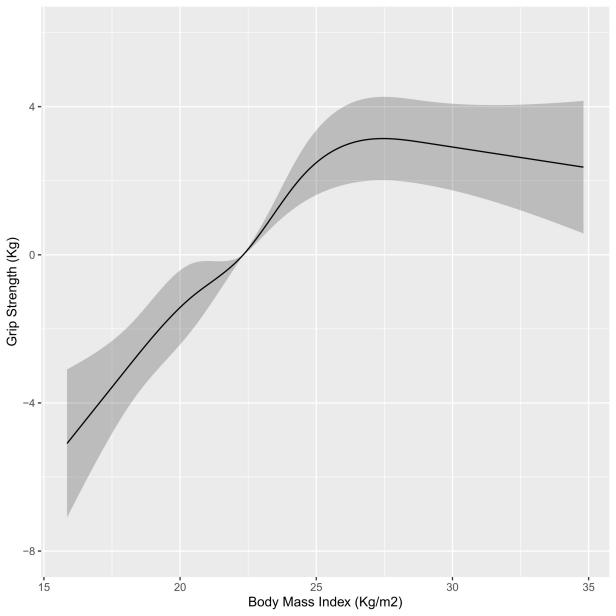


**A**

**B**

Figure S4 Nonlinear associations between baseline BMI and follow-up GS for females (A) and males (B).
